# Brain organoids: Establishment and application

**DOI:** 10.3389/fcell.2022.1029873

**Published:** 2022-11-23

**Authors:** Hao Chen, Xin Jin, Tie Li, Zhuang Ye

**Affiliations:** ^1^ Department of Neurovascular Surgery, First Hospital, Jilin University, Changchun, China; ^2^ Department of Oncology and Hematology, Second Hospital, Jilin University, Changchun, China; ^3^ Department of Rheumatology, First Hospital, Jilin University, Changchun, China

**Keywords:** organoid, brain organoids, pluripotent stem cells, neurological diseases, 3D culture, brain disease modeling, drug screening, organ transplantation

## Abstract

Brain organoids are produced by the differentiation of pluripotent stem cells under three-dimensional culture conditions by adding neurodevelopment-related regulatory signals. They are similar to the cell composition and anatomical structure of the brain, and can reflect the developmental process of the brain, as well as their physiology, pathology, and pharmacology. Brain organoids are good models to study human brain development and brain-related diseases *in vitro*. Here, we mainly focus on the construction of brain organoids and review the application of brain organoids in disease modelingand drug screening.

## Introduction

An organoid is a three-dimensional (3D) cell culture product, which is an organ-specific product obtained by inoculating stem cells in matrigel or basement membrane extract under the action of a specific cytokine mixture (Sato et al., 2009; Shariati et al., 2021). Organoids often contain multiple types of organ-specific cells and can reproduce some organ functions *in vitro*. Compared with traditional cell models, organoid models have more stable genetic and phenotypic characteristics during *in vitro* culture and more abundant cell types (Takahashi, 2019; Li et al., 2020; Tang et al., 2022).

The brain is the largest and most complex organ with complex neural activity (Northcutt and Kaas, 1995). The power of the brain, especially in humans, covers movement, sensation, vision, hearing, and more advanced brain functions such as consciousness and memory (Taverna et al., 2014). The complexity of brain structure and function, especially the unique functional division of the human brain, poses challenges to brain research. Due to ethical confine and human brain tissue source constraint, researchers have traditionally used animal models to study human brain development. Therefore, the research on human brain development often stays at the common characteristics of mammals and other vertebrates. However, the unique structure and function of human brain development makes it hard to show the development feature of the human brain in animal models, especially in many neurodegenerative diseases highly related to gene variation, such as schizophrenia, autism spectrum disorder (ASD), Alzheimer’s disease (AD), Parkinson’s syndrome (PD), *etc.*

The development of stem cell technology enables researchers to use human-induced pluripotent stem cells (hiPSC) to induce brain-like tissues and organs from a 3D perspective (Takahashi and Yamanaka, 2006; Lancaster et al., 2013). Brain organoids are similar to the brain in cell composition and anatomical structure, and can simulate the developmental process of the brain, reflecting the physiological, pathological, and pharmacological characteristics of the brain (Chiaradia and Lancaster, 2020; Sharf et al., 2022). In the past few years, brain organoid technology has been realized from unguided whole-brain organoids, gradually to the cortex, midbrain, hippocampus, cerebellum, spinal cord, and other brain organoids with regional characteristics (region-specific brain organoids) and achieved vascularization of brain organoids (Jo et al., 2016; Qian et al., 2016; Monzel et al., 2017; Qian et al., 2018; Cakir et al., 2019; Zagare et al., 2021; Cakir et al., 2022).

Neurodevelopmental disease (NDD) refers to the abnormal development of the nervous system caused by hereditary or acquired etiology, resulting in brain dysfunction, including intellectual disability, autism spectrum disorder, attention deficit hyperactivity disorder, and other diseases. Analyzing the pathogenic mechanism of neurodevelopmental diseases has always been one of the key topics in neurobiology. But due to the ethics restrain, human and non-human primate brain tissue shortage, even though a small number of patient tissues can be obtained, it can only reflect the disease in the terminal stage, the occurrence and development of the disease cannot be analyzed. Therefore, the knowledge and understanding of human brain developmental diseases are mainly derived from studies on rodents. However, the complex structural and functional partitions unique to the human brain cannot be fully reproduced by animal models. The development of stem cell technology, especially the establishment of induced pluripotent stem cells, provides an ideal human cell model for decoding the pathogenesis of neurodevelopmental diseases. Reprogramming of patient somatic cells with disease genes into induced pluripotent stem cells (iPSCs) followed by differentiation into various types of nerve cells has been widely used in the study of various neurological diseases. However, neurological disease phenotypes are highly heterogeneous, including abnormalities in brain structure such as brain size, and problems with synaptic activity. For this complexity, traditional two-dimensional (2D) neural culture can only provide limited insights. Subsequently, a 3D brain organoid culture protocol was pioneered by improving the 2D neural culture method, opening new horizons for the study of neurodevelopmental diseases.

The generation of brain organoid technology not only makes up for the shortcomings of traditional 2D cell culture that cannot simulate the complex structure of brain tissue and the *in vivo* microenvironment, and is difficult to reproduce the complex phenotypes of neurological diseases, but also breaks through the lack of human-specific genetic characteristics, brain regions, and functions, difficulties in comprehensively simulating the development of the human brain and the limitations of disease occurrence and development. It is an important tool to study human brain development and evolution *in vitro*, to explore the interaction of different brain regions, brain, and other organs, and carry out disease simulation and drug screening *in vitro*. This article will review the history of the establishment and development of brain organoids, introduce the progress of brain organoids in the exploration of brain development, neurological disease simulation, and drug screening, and analyze possible future directions.

## Construction of brain organoids

In 1981, researchers isolated embryonic stem cells from the inner cell mass of mouse blastocysts (Evans and Kaufman, 1981). In 1998, Thomson et al. (1998) successfully isolated human embryonic stem cells and further differentiated them into various types of tissue cells, including neuroepithelial and embryonic ganglion cells. In 2006, Takahashi and Yamanaka (2006) induced mouse fibroblasts to generate pluripotent stem cell iPSCs with stem cell properties by overexpressing transcription factors Sox2, Klf4, Oct3/4, and c-Myc. In 2007, similarly, they overexpressed four key transcription factors including Oct3/4 (Pou5f1), Sox2, Klf4, and c-Myc in differentiated and mature somatic cells to make them return to a pluripotent state and obtain human-induced pluripotent stem cells (hiPSCs) (Takahashi et al., 2007). Compared with embryonic stem cells, hiPSCs are easy to obtain and have a wide range of sources. They can be obtained from the somatic cells of patients, avoiding ethical issues and immune rejection, and providing a new way for individual precision treatment. The earliest *in vitro* model used for brain research was the embryoid body (EB)-derived neural rosettes (NR). Neural rosette is a human neural tube-like structure that appears during the differentiation of hiPSCs into cerebral organoids and is the basis for the differentiation of various nerve cells. The further development of hiPSCs technology has promoted the generation of induced pluripotent stem cell neurospheres and three-dimensional neuroepithelial tissue, which can reflect the genetic characteristics of the human brain to a certain extent, but they do not form a complete and complex brain structure, and they lack the coordination between different sub-subsections of the brain.

However, traditional neuronal diseases are simulated using neurons differentiated and developed from human pluripotent stem cells (hPSCs), which is also a 2D culture-based system (Avior et al., 2016). Although various types of neurons with stable homogeneity and high purity can be generated by the current 2D differentiation system (Tyler, 2012), such as motor neurons, telencephalic excitatory neurons, and midbrain dopaminergic neurons (Subramanian et al., 2009), the 2D cultured cell model has certain shortcomings, it can neither simulate the microenvironment composed of various regulatory factors and endogenous signals in the three-dimensional space of the brain (Aloysious and Nair, 2014) nor can it simulate the interaction between cells in the three-dimensional space. The function of different types of cells in different brain regions in neurological diseases cannot be well simulated. Therefore, the establishment of a 3D culture system came into being.

In 2013, Lancaster et al. (2013) for the first time used hiPSCs to induce differentiation into brain organoids, and study microcephaly. They induced and directionally differentiated hiPSCs to produce an embryoid body structure with inner, middle, and outer germ layers, and then passed through the neuroectoderm and neuroepithelial layers, and finally formed a structure similar to the early embryonic cerebral cortex which can reflect the developmental process of the human brain in the early embryonic stage. Qian et al. (2016) applied microbioreactors to maintain Zika virus (ZIKV)-treated brain region-specific organoids. Kirwan et al. (2015) also successfully constructed an organoid model of the cerebral cortical neural network using human iPSC lines, which can simulate the development and function of the cortical network. Pham et al. (2018) cultured human iPSCs into cerebral organoids, differentiated iPSCs from the same source into endothelial cells (EC), and re-embedded the organoids matrigel with 250, 000 endothelial cells after 3D culture for some time, resulting in vascularized brain organoids. Dang et al. (2016) performed RNA sequencing on organoids and found that the gene expression profiles of brain organoids cultured to day 30 were very similar to those of human fetal brain samples from 8 to 9 weeks of gestation. Compared with research methods such as animal models and cell culture, brain organoids can simulate the developmental process and structural characteristics of the early human embryonic brain to a certain extent *in vitro*, and can better maintain human-specific genotypes and protein expression levels. With the advancement of bioengineering technology, the development of pluripotent stem cell-derived 3D brain organoid technology has become more mature, and its application prospects have become broader and broader. Taken together, pluripotent stem cell-derived 3D brain organoid technology can be used to break through the current bottleneck in the study of brain development and neurological diseases and has great potential as a model for brain development and neurological disease research.

## Brain organoids and disease modeling

Current research on neurological diseases mainly uses animal models. However, the genetic background, and physiological and pathological characteristics of model animals are different from those of humans. So it is difficult to simulate the occurrence and development of diseases in human brain truly and comprehensively. As an effective complement, human brain organoids provide new tools for modeling human brain development, psychiatry, and degenerative diseases, as well as for drug screening and gene therapy.

### Autism spectrum disorder

Autism spectrum disorder (ASD) is a brain developmental disorder characterized by language impairment, social difficulties, and repetitive stereotyped behaviors (Lord et al., 2018; Lord et al., 2020). Mariani et al. (2015) induced iPSCs of autistic patients with macrocephaly phenotype into telencephalic organoids, and found that in the early stage of brain organoid development, the cell cycle of neural progenitor cells is shortened, and the increase of GABAergic neurons leads to excitatory/inhibitory neural imbalance, the mechanism is caused by the abnormality of FOXG1 gene and its downstream molecules. One group constructed a sliced neocortical organoid (SNO), which can avoid the problem of cell death in the organoid due to long-term hypoxia and lack of nutrients, thereby generating a larger progenitor cell area and neural layer (Qian et al., 2020). Using this organoid model, it was found that the mutation of the autism susceptibility gene DISC1 leads to the impairment of WNT/β-catenin signaling and the disorder of cortical neuron fate differentiation, which leads to the abnormality of lamellar markers SATB2, TBR1, ROB, and CITP2. These defects can be rescued by correcting the mutation using gene-editing methods. Another group constructed iPSCs with heterozygous deletion of CHD8 by CRISPR-Cas9 and induced them into brain organoids (Wang et al., 2017). By comparing the transcriptome data of brain organoids with CDH8 deletion and isogenic control, they found that CHD8 regulates ASD-related genes such as TCF4, AUTS2, differential genes are also involved in neurogenesis, WNT/β-catenin signaling pathway, GABA neuron differentiation, *etc.* Another group found that the volume of brain organoids with RAB39B mutation increased, and the excessive proliferation of neural progenitor cells led to the thickening of the SOX2 positive ventricular zone (VZ), leading to differentiation disorder. Also, the lack of RAB39B causes the PI3K-AKT-mTOR signaling pathway excessively activated. And inhibiting the PI3K-AKT-mTOR signaling pathway can rescue the autism phenotype (Zhang et al., 2020).

### AD

AD, commonly known as senile dementia, is a neurodegenerative disease characterized by memory impairment, cognitive dysfunction, and behavioral disorders with classical pathological features of β-amyloid (amyloid β-protein, Aβ) deposition and neurofibrillary tangles (neurofibrillary tangles, NFTs) (Scheltens et al., 2021; Pleen and Townley, 2022). However, Existing AD transgenic mouse models can only show Aβ-induced synaptic and memory deficits but cannot fully reflect the pathological characteristics of neurofibrillary tangles (Higgins and Jacobsen, 2003; McGowan et al., 2006; Chin, 2011). Moreover, neurons derived from AD patients show high levels of Aβ toxicity and tau protein phosphorylation but are also unable to replicate amyloid-β plaques and neurofibrillary tangles (Choi and Tanzi, 2012; Israel et al., 2012). Taken together, above mentioned methods cannot fully mimic the AD disease which limits the mechanism exploration and drug discovery. As technology advances, people begin to use organoids as models to study AD. In 2014, one group, for the first time, constructed the AD brain organoid model induced by human neural stem cells stably transfected with APP and PS1 mutant genes. This model reproduced the pathological features of AD and verified the amyloid hypothesis of AD. That is, excessive accumulation of Aβ can lead to neurofibrillary tangles composed of an aggregation of hyperphosphorylated tau protein (Choi et al., 2014). Later, another study conducted 2D and 3D differentiation models of AD-derived iPSCs at the same time, and found different therapeutic effects of the same drug in 2D and 3D models, suggesting that the 3D system can better simulate cell-cell interactions and the microenvironment of which is more similar to that *in vivo* (Lee et al., 2016). Another group established an AD co-cultured brain organoid model composed of neurons, astrocytes, and microglia, which can recruit microglia, simulate axonal damage, and achieve amyloid aggregation and accumulation of phosphorylated tau protein (Park et al., 2018). This system provides more physiologically relevant systems for *in vitro* human AD culture models to explore key pathological features of AD. Another study found that APOE4 may be a potential target for the treatment of AD by using brain organoids (Zhao et al., 2020). In addition, several promising drugs screened in AD mouse models failed to improve cognition in late-stage clinical trials. These together suggested that 3D brain organoids can not only provide the possibility to study the complex pathological mechanisms of human brain diseases but also can be promising new platforms for the discovery of drugs for neurodegenerative diseases.

### Glioblastoma

Glioblastoma (glioblastoma multiforme, GBM) is the most common and aggressive primary malignant brain tumor (Ricard et al., 2012; Omuro and DeAngelis, 2013; Wirsching et al., 2016; Lapointe et al., 2018). Despite decades of intensive research, the average survival time of patients remains at 12–15 months (Aldape et al., 2019). One big challenge in advancing GBM therapy is the current lack of ideal models to study the properties of human GBM, especially the invasion of surrounding brain tissue. Traditional *in vitro* culture models, whether monolayer culture or tumor sphere culture, may take a lot of time to establish and use exogenous epidermal growth factor, basic fibroblast growth factor, and/or serum to serially pass tumor cells in a clonal expansion fashion, which is not conducive to maintaining the various cell subtypes and key driver gene expression of the parent tumor, and lack of organoid histological features and interaction between tumor and normal tissue (Lee et al., 2006; Schulte et al., 2012). An alternative way is the PDX (patient-derived tumor xeno-graft) model in which isolated primary tumor cells are injected directly into mice which are thought to better preserve these important features of GBM (Giannini et al., 2005). In addition, genetically engineered mouse models are also not ideal models due to the divergences between humans and mice, especially in the brain. Therefore, we need more accurate models that can not only reproduce the tumor phenotype and the complex tumor microenvironment but also can support our detailed study of the mechanism of tumor occurrence and development.

Organoid models that have emerged in recent years have made up for the above shortcomings. At present, various types of organoids have been successfully cultured *in vitro*, including brain organoids (Lancaster et al., 2013; Paşca et al., 2015). And organoids have been applied to models of various cancers, including liver, breast, pancreatic, prostate, bladder, ovarian, and gastrointestinal cancers, and have also been used for the exploration of tumor development and drug resistance mechanisms (Gao et al., 2014; Phillips, 2014; Khan et al., 2021). Therefore, the successful establishment of organoids is of great significance for GBM research.

In 2016, one group developed a tumor organoid culture system directly derived from GBM specimens (Hubert et al., 2016). In this system, tumor cells are suspended in matrigel, which can reach a maximum of about 3–4 mm after 2 months of culture. Organoid growth slowed significantly in subsequent cultures, but GBM organoids remained stable and viable without passage over a year of continuous culture. These organoids reproduce the features of *in vivo* tumor hypoxic gradients and tumor stem cell heterogeneity. Although this organoid model is promising, further characterization and validation across multiple GBMs are still required. And the construction success rate has not been determined and may be patient specific. The number of model build is relatively low to moderate throughput, and the time required to set up the culture is long. Recently, another group reported a new and faster method for GBM organoid establishment (Jacob et al., 2020). This method does not separate and digest tumor specimens, but slices the specimens into pieces around 1 mm, and establishes organoids without matrigel and serum, without the addition of EGF and FGF. Using histopathology, immunohistochemistry, single-cell transcriptomics, mutational analysis, and other methods, the researchers confirmed that the organoid can accurately reproduce the histological features, cell diversity, gene expression, and mutational characteristics of the parental tumor (Jacob et al., 2020). GBOs (glioblastoma organoids) reproduce tumor tissue architecture rather than the brain, more closely reproduce the complexity and heterogeneity of the primary tumor, and have great potential for studying GBM biology and predicting treatment response. Their main limitation is that they need to be re-transplanted back into the body to study their interaction with healthy brain tissue. Another interesting study reported that immunodeficient mice began to die within a few months after injecting organoid-derived tumor cells unilaterally into the hippocampus (Ogawa et al., 2018). They found that organoid-derived tumor cells spread along blood vessels, and HE staining showed extensive invasiveness and nuclear pleomorphism, similar to the biological features of human GBM. In addition, areas of tumor cell foci showed extensive Ki-67 expression, strong CD31 staining demonstrated that the tumor was highly angiogenic, and displayed GBM tumor stem cell positive markers such as SOX2 and GFAP. This study demonstrated that organoid-derived GBMs possessed full oncogenic potential and characteristics of human tumors. In another study, the researchers co-cultured patient-derived GBM spheroids with brain organoids derived from early mouse embryonic stem cells (da Silva et al., 2018). They found that GBM spheroids spontaneously attached to organoids and subsequently fused and invaded brain tissue. Compared with control neural progenitor cells, GBM cells have greater migratory capacity and infiltrate the inner layers of brain organoids more efficiently. Taken together, organoids mimic glioblastoma invasion with time advantages, high engraftment efficiency, strong invasiveness, and retention of key driver mutation expression. This provides a good platform for further research on the underlying mechanism and subsequent treatment of glioma.

### Sandhoff disease

Sandhoff disease is a lysosomal aggregation disorder in which GM2 gangliosides are deposited in the brain due to lack of hexosaminidase A (HexA) and hexosaminidase B (HexB) activity (Parenti et al., 2015; Marques and Saftig, 2019; Martina et al., 2020). At the same time, the final products of β-hexosamine, glycolipids, glycoproteins, and oligosaccharides are also deposited in the brain and internal organs. One group constructed brain organoids using iPSCs derived from infant fibroblasts with Sandhoff disease and used CRISPR/Cas9 technology to create isogenic (HEXB-corrected) controls (Allende et al., 2018). They found that organoids from Sandhoff disease showed GM2 gangliosides accumulation, whereas the controls did not. Diseased organoids also exhibited increased volume and cell proliferation compared to their isogenic controls. Transcriptomic analysis also reveals impaired development of Sandhoff disease organoids. It can be seen that as a model of early developmental diseases, brain organoids can well represent the pathological characteristics of Sandhoff disease, providing an important research method for such rare diseases.

### Fragile X syndrome

Fragile X syndrome (FXS) is an intellectual disability syndrome caused by mutations in the FMRP gene on the X chromosome (Salcedo-Arellano et al., 2020). Researchers constructed FMR1 gene-truncated iPSCs by CRISPR/Cas9 and simulated some phenotypes of FXS *in vitro* by 2D neural culture and 3D brain organoids, respectively (Brighi et al., 2021). Compared with isogenic control brain organoids, FXS brain organoids exhibited larger size and more GFAP-positive glial cells, which is consistent with the observed effect of the FMR1 gene on neural progenitor cell proliferation under 2D culture conditions, suggesting that the brain organoid model can be used to explore the pathological mechanism of X-linked intellectual disability and provide a new platform for the treatment of such diseases.

### Lissencephaly

Lissencephaly, also known as the smooth brain, is a neuronal migration disorder (Koenig et al., 2021). Among them, Miller-Dieker syndrome (MDS) is the most serious one, which is caused by a massive loss of heterozygosity on human chromosome 17p13.3, involving PAFAH1B1, YWHAE, and other genes (Toyo-oka et al., 2003; Liu et al., 2021). Due to the absence of oRG cells in the cortex of rodents, the phenotype of Pafah1b1 mutant mice is much weaker than that of PAFAH1B1 mutant human patients (Shitamukai et al., 2011). In a previous study, people used iPSCs from patients with MDS to culture and analyze brain organoids, and found that in the stages of neuroepithelial cell expansion, neuronal migration and oRG progenitor cell mitosis, neural stem cells in MDS organoids were massively apoptotic, and oRG cells exhibit a delay in mitosis, leading to defects in neuronal migration (Bershteyn et al., 2017). Another group found that MDS organoids exhibited premature neurogenesis, decreased cortical expansion rate, significantly reduced organoid tissue volume, and accompanied by ventricular zone radial glia cells transition from symmetric to asymmetric cell division, the N-cadherin/β-catenin/WNT signaling pathway is inhibited, and administration of an agonist of WNT signaling can alleviate the MDS phenotype (Iefremova et al., 2017).

### MCPH (autosomal recessive primary microcephaly)

Autosomal recessive primary microcephaly is a neurodevelopmental disorder caused by autosomal aberrations, which is mainly characterized by small brains, especially cerebral cortical changes (Faheem et al., 2015; Naveed et al., 2018). Among the genes known to be associated with microcephaly, CDK5 regulatory subunit-associated protein 2 (CDK5RAP2) regulates the replication process of centrosome proteins, and loss or mutation of CDK5RAP2 can cause microcephaly disease (Hassan et al., 2007; Abdullah et al., 2017; Sukumaran et al., 2017). However, previous studies demonstrated that mice are not an ideal model for mimicking MCPH, because they observed that the cerebral cortex of CDK5RAP2 mutant mice was not significantly smaller than that of human patients (Lizarraga et al., 2010). To overcome the shortage, one group constructed brain organoids derived from iPSCs of microcephaly patients carrying CDK5RAP2 mutations (Lancaster et al., 2013). Compared to controls, patient-derived organoids displayed smaller neuroepithelial regions and smaller overall dimensions, well mimicking the symptoms of microcephaly patients. Based on this research, many studies using brain organoids to explore the mechanism of Zika virus (ZIKV)-induced microcephaly have been reported. ZIKV infection caused an overall reduction in the size of organoids, ZIKV also induced apoptosis in neural precursor cells, attenuated precursor cell proliferation, and increased the size of the lumen of the ventricular structure (Cugola et al., 2016; Dang et al., 2016; Garcez et al., 2016; Qian et al., 2016; Sutarjono, 2019). These results are consistent with a clinical case report describing the lumen of enlarged ventricular structures observed in ZIKV-infected human fetal brains (Driggers et al., 2016). It can be seen that the brain organoid model can help researchers to explore the mechanism of neural and brain development.

### Schizophrenia

Brain organoids, as an emerging *in vitro* model, have played an important role in the study of psychiatric disorders. Schizophrenia is a highly heritable mental disorder, and a study based on DNA whole-genome sequencing of schizophrenia patients found that 15q11.2 gene copy number variation is one of its risk factors (Levinson et al., 2011; Malhotra and Sebat, 2012). Yoon et al. (2014) compared iPSCs-derived human neural precursor cells with 15q11.2 copy number microdeletion with those without deletion and found that 15q11.2 copy number microdeletion can lead to defects in neural precursor cell adhesion junctions and apical polarity. Disrupted-in-schizophrenia 1 (DISC1) is a potential susceptibility gene for many psychiatric disorders, including schizophrenia, depression, and bipolar disorder. Although there are many studies on DISC1, how does DISC1 protein Interacting with other proteins to affect brain function is rarely reported. The researchers induced the differentiation of pluripotent stem cells from schizophrenia patients with DISC1 mutations into brain organoids and found important results: DISC1 and nuclear distribution protein nudE-like 1 (NDEL1) binding can regulate neural Stem cell division, DISC1 mutations cause retarded nerve cell division in schizophrenia (Ye et al., 2017). Other group also used similar technology to study this (Srikanth et al., 2015).

## Brain organoids and drug screening

Brain organoids are expected to become an efficient platform for drug development, screening, and testing. Drug safety evaluation is an important reference for drugs to enter the clinical stage. Brain organoids are expected to become important models for drug screening and reduce the testing burden of clinical trials due to their high molecular and structural similarity to the source tissue. Zika virus research with brain organoid technology not only reveals the relationship between Zika virus outbreaks and the incidence of congenital microcephaly but also has great potential for drug testing, including the preparation of potential Zika virus antiviral drugs. In addition, brain organoids are also widely used in other common drug screenings. Some studies have used a brain organoid model constructed by neural progenitor cells, glial cells, and neurons to explore the effects of METH on the human brain (Dang et al., 2021). The study found that in METH-treated brain organoids, cytokine CXCL8 was up-regulated, and neuroinflammation-related gene expression was also up-regulated, indicating that brain organoids are immune responsive, and METH treatment triggered neuroinflammation in brain organoids. More excitingly, neuronal organoids reproduced the characteristics of the blood-brain barrier during co-culture with endothelial cells, thus providing the possibility for screening drugs targeting the central nervous system (Phan et al., 2017). Therefore, brain organoids have great potential in testing drug effects and side effects due to their high reduction of the structure and function of human organs.

## Conclusion and perspectives

Since its establishment, *in vitro* organoid culture technology has experienced rapid development and has shown strong application value. In general, organoids can well mimic the corresponding tissues in patients at the gene level and morphological characteristics, are also suitable for high-throughput drug screening and provide a research model for personalized treatment of diseases.

Human brain organoids have attracted great interest in recent years due to their potential to study human brain development and neuropathology without the constraints of animal models. The development of 3D brain organoid technology is less than 10 years and is still in its infancy. In terms of their cellular and molecular composition, the current architecture of brain organoids can mimic the second-trimester human fetal brain. However, since cerebral organoids lack vascular circulatory system, they mainly rely on free diffusion for oxygen exchange and nutrient uptake from the culture medium. When cultured for a long time *in vitro*, cells in the middle of the organoid undergo massive apoptosis due to a lack of oxygen and nutrients. Therefore, the establishment of an improved circulatory system of brain organoids is the general trend. In addition, current brain organoid technologies still suffer from significant limitations, especially in the functional assessment of neural network activity and cellular interactions, and neural circuit functions relevant to neurodevelopmental and neuropsychiatric pathologies (Quadrato et al., 2017).

In summary, although the current brain organoid culture system still has technical defects, it does not have the 3D structure and complex functions of human natural organs. However, it is undeniable that the 3D brain organoid model has brought great progress to human research on brain development and disease mechanisms (Table 1). The construction and application of brain organoids will still be the focus of attention in the field of life medicine in the future. And the application of organoid transplantation to replace drug therapy to cure neurological diseases is still an important development direction of precision medicine in the future. For example, one group successfully recoded human and mouse fibroblasts to form sensory ganglion organoids, and the induced sensory neurons had electrophysiological properties and calcium ion response properties (Xiao et al., 2020). In the future, the induced sensory ganglion organoids may be widely used as an important cell source for replacement therapy of damaged or degenerated sensory neurons. As an emerging biological culture technology, brain organoids have great research potential and application value in the study of human brain development, disease mechanism, tissue replacement therapy, and drug screening.

**TABLE 1:** Application of brain organoids: diseases modelling and drug screening.

Application	Gene	References
ASD	FOXG1	Mariani et al. (2015)
ASD	DISC1	Qian et al. (2020)
ASD	CHD8	Wang et al. (2017)
ASD	RAB39B	Zhang et al. (2020)
AD	APP, PS1	Choi et al. (2014)
AD	—	Lee et al. (2016); Park et al. (2018)
AD	APOE4	Zhao et al. (2020)
GBM	—	Hubert et al. (2016; Jacob et al. (2020); Ogawa et al. (2018); da Silva et al. (2018)
Sandhoff	—	Allende et al. (2018)
FXS	FMR1	Brighi et al. (2021)
Lissencephaly		Bershteyn et al. (2017); Iefremova et al. (2017)
MCPH	CDK5RAP2	Lancaster et al. (2013)
ZIKV-induced microcephaly		Cugola et al. (2016); Dang et al. (2016); Garcez et al. (2016); Qian et al. (2016); Sutarjono, (2019)
Schizophrenia	15q11.2 copy number microdeletion	Yoon et al. (2014)
Schizophrenia	DISC1	Ye et al. (2017)
Drug screening		Phan et al. (2017); Dang et al. (2021)
